# Modes of Large-Scale Brain Network Organization during Threat Processing and Posttraumatic Stress Disorder Symptom Reduction during TF-CBT among Adolescent Girls

**DOI:** 10.1371/journal.pone.0159620

**Published:** 2016-08-09

**Authors:** Josh M. Cisler, Benjamin A. Sigel, Teresa L. Kramer, Sonet Smitherman, Karin Vanderzee, Joy Pemberton, Clinton D. Kilts

**Affiliations:** Brain Imaging Research Center, Department of Psychiatry, University of Arkansas for Medical Sciences, Little Rock, Arkansas, 72205, United States of America; University of Stellenbosch, SOUTH AFRICA

## Abstract

Posttraumatic stress disorder (PTSD) is often chronic and disabling across the lifespan. The gold standard treatment for adolescent PTSD is Trauma-Focused Cognitive-Behavioral Therapy (TF-CBT), though treatment response is variable and mediating neural mechanisms are not well understood. Here, we test whether PTSD symptom reduction during TF-CBT is associated with individual differences in large-scale brain network organization during emotion processing. Twenty adolescent girls, aged 11–16, with PTSD related to assaultive violence completed a 12-session protocol of TF-CBT. Participants completed an emotion processing task, in which neutral and fearful facial expressions were presented either overtly or covertly during 3T fMRI, before and after treatment. Analyses focused on characterizing network properties of modularity, assortativity, and global efficiency within an 824 region-of-interest brain parcellation separately during each of the task blocks using weighted functional connectivity matrices. We similarly analyzed an existing dataset of healthy adolescent girls undergoing an identical emotion processing task to characterize normative network organization. Pre-treatment individual differences in modularity, assortativity, and global efficiency during covert fear vs neutral blocks predicted PTSD symptom reduction. Patients who responded better to treatment had greater network modularity and assortativity but lesser efficiency, a pattern that closely resembled the control participants. At a group level, greater symptom reduction was associated with greater pre-to-post-treatment increases in network assortativity and modularity, but this was more pronounced among participants with less symptom improvement. The results support the hypothesis that modularized and resilient brain organization during emotion processing operate as mechanisms enabling symptom reduction during TF-CBT.

## Introduction

Early life assaultive violence exposure is a potent risk factor for pediatric posttraumatic stress disorder (PTSD), particularly among girls [1,2]. Left untreated, PTSD is often chronic and associated with significant morbidity and poor quality of life across the lifespan [3,4]. The current gold standard treatment for pediatric PTSD is Trauma-Focused Cognitive-Behavioral Therapy (TF-CBT) [5–7], which is a manualized treatment typically delivered in 12–16 weekly sessions and includes modules addressing psychoeducation about trauma and PTSD; parenting skills; affect regulation skills; and developing a narrative of the traumatic event and cognitive processing of associated thoughts and feelings. Numerous clinical trials have demonstrated efficacy for TF-CBT in reducing PTSD symptoms, depression, anxiety, and behavior problems among traumatized youth [8].

Despite the replicated efficacy of TF-CBT, treatment response is variable and tends to demonstrate a moderate effect size [8]. For example, in a recent TF-CBT clinical trial, the mean decrease in total number of PTSD symptom from pre-to-post treatment was 3.3 with a standard deviation of 3.48, indicating significant individual differences in treatment response [7]. The purpose of the present study was to identify modes of functional organization of neural processing networks as predictors of individual variation in treatment response towards the larger goal of elucidating for whom TF-CBT is most likely to work and through what brain organization principles it produces clinical outcomes.

Contemporary neuroimaging research is moving away from functional segregation analyses and towards computational approaches that model the brain as a distributed network of information processing [9–11]. These approaches frequently employ concepts from graph theory and model the brain as a graph composed of nodes (brain regions or vertices) and edges (connections between nodes). Graph-theory-based analyses of human brain functional connectivity have been used to demonstrate important large-scale organizing principles of the human brain, including small world properties, high communication efficiency, and resilience to targeted node attacks [12–14]. One graph theory concept that is hypothesized to be a key organizing principle of most complex systems is modularity [15]. Modularity refers to the degree to which a network can be subdivided into discrete and functionally specialized modules (or communities), and a modularized network is characterized by greater within-module connectivity relative to between-module connectivity [16]. Modular organizational properties may emerge out of evolutionary and developmental demands to simultaneously minimize connection costs and average path lengths while maximizing network adaptive value and dynamic stability [13,17]. Modular networks are more adaptive to changing environmental demands and more resilient to structural perturbations of the network [13,18]. Prior research demonstrates decreased modular brain organization among individuals high in neuroticism [19] and diagnosed with schizophrenia [20], and that modular organization of the human brain becomes more sophisticated across development [21], thus demonstrating associations between the degree of modularity of functional brain networks and clinical, personality, and neurodevelopmental processes.

Related graph theoretical concepts pertaining to optimal global network functional organization include assortativity and global efficiency. Assortativity characterizes the degree to which highly connected nodes tend to connect to other highly connected nodes [22]. Networks with positive assortativity coefficients tend to be more resilient to targeted node insults due to having mutually interconnected central nodes (i.e., hubs), whereas networks with negative assortativity coefficients are vulnerable to insults due to their having relatively disconnected hubs [23,24]. Global efficiency refers to the average inverse shortest path length (i.e., routes of information flow between nodes) within a network and is a general measure of network integration, with higher global efficiency representing a more integrated network [22].

Recent research had demonstrated altered large-scale brain organization in PTSD. Pediatric PTSD has been linked with a greater clustering coefficient (i.e., fraction of a node’s neighbors that neighbors of each other, averaged across the network) yet with a higher average path length during resting-state [25]. In a similar analysis, adults with PTSD demonstrated increased small-worldness of brain networks during resting-state, characterized by higher clustering coefficients and lower path lengths [26]. Among adults exposed to combat, higher re-experiencing symptoms were related to lower network density (i.e., fewer network connections) during resting-state[27]. The current analysis adds to this early and growing literature by examining large-scale brain organization during task and linking network properties with treatment outcome. As a relevant example of the role of global network organization as predictors of treatment outcome, obsessive compulsive disorder symptom reduction during pharmacological treatment was shown to be significantly correlated with increases in global small-world brain network organization and modularity [28].

Here, we investigate whether particular patterns of large-scale brain network organization, characterized using the global network indices of modularity, assortativity, and global efficiency, similarly operate as mechanisms predicting PTSD symptom reduction during TF-CBT. Support for this hypothesis would suggest novel models of the brain mechanisms by which TF-CBT variably works and for whom it is most likely to work. We focused on a pediatric PTSD sample homogenous with respect to age (11–16), sex (all girls), and trauma exposure (all exposed to assaultive violence). Given the centrality of emotion processing to neurocircuitry models of PTSD [29,30], we characterized global network functional organization patterns during an emotion processing task.

## Method

### Participants and assessments

Thirty-four adolescent girls, aged 11–16, meeting DSM-IV criteria for PTSD, having a positive history of assaultive violence exposure, and having a consistent caregiver with whom to participate in treatment, were enrolled in the study and began TF-CBT. Twenty adolescent girls completed all 12 sessions of TF-CBT and had usable imaging data at pre- and post-treatment (e.g., no excessive head motion, see below). Participants were recruited through networking with local outpatient clinics, child advocacy centers, schools, juvenile justice system, churches, and community organizations. No participant had previously received TF-CBT. Exclusion criteria consisted of MRI contraindications (e.g., internal ferromagnetic objects), psychotic symptoms, lack of a consistent caregiver, and presence of a developmental disorder. Concurrent psychotropic medication was not exclusionary. Demographic and clinical characteristics of the sample are provided in Table 1. Adolescents provided written assent and a caregiver/legal guardian provided written informed consent. This study was conducted with the University of Arkansas for Medical Sciences Institutional Review Board approval of all study procedures.

**Table 1:** Demographic, clinical characteristics, and treatment response of the samples.

	All Patients (n = 20)	Large Treatment Response (n = 10)	Small Treatment Response (n = 10)	P value of group difference
Variable	Mean/frequency (SD)	Mean/frequency (SD)	Mean/frequency (SD)	
Age	13.75 (1.8)	14.4 (1.8)	13.1 (1.7)	.66
Verbal IQ	92.6 (13.32)	93.7 (15.8)	91.4 (11.1)	.71
Ethnicity	35% Caucasian	40% Caucasian	30% Caucasian	.66
	55% African American	60% African American	50% African American	
	10% Biracial	0% Biracial	20% Biracial	
Total number of types of assaults	5.9 (4.22)	5.7 (3.3)	6.1 (5.1)	.84
Psychotropic Medication	50%	30%	70%	.40
Pre-Treatment UCLA PTSD Index	37.3 (17.88)	39.83 (17.3)	34.82 (17.87)	.49
PTSD symptom reduction slope	-.95 (.70)	-1.44 (.51)	-.46 (.46)	< .001
SMFQ	12.1 (8.5)	12.3 (8.9)	11.9 (8.6)	.92
# comorbid diagnoses	3.9 (2.3)	2.1 (2.0)	3.6 (2.5)	.15
Bipolar Disorder	0%	0%	0%	-
Major depression	60%	60%	60%	-
Anxiety disorder	45%	40%	50%	.67
Alcohol Use Disorder	10%	10%	10%	-
Substance Use Disorder	15%	10%	20%	.56

Note. SMFQ = Short mood and feelings questionnaire.

As part of a separate study, a cohort of 15 healthy adolescent girls, aged 12–16 (mean = 14.27; SD = 1.28), with no history of interpersonal violence, no current mental health disorders, and no current psychotropic medication were additionally recruited and participated in the identical emotion processing task during fMRI.

PTSD participant’s pre- and post-treatment mental health was assessed with the MINI-KID [31], a structured clinical interview for most Axis I disorders found in childhood and adolescence. Assaultive trauma histories were characterized using the trauma assessment section of the National Survey of Adolescents (NSA) [1,2], a structured interview used in prior epidemiological studies of assault and mental health functioning among adolescents that uses behaviorally specific dichotomous questions to assess sexual assault, physical assault, severe abuse from a caregiver, and witnessed violence. A trained female research coordinator with several years of experience with structured clinical interviews completed the MINI and NSA interviews with participants under the supervision of a licensed clinical psychologist.

The pre- and post-treatment assessment also included measures of verbal IQ (receptive one word picture vocabulary test [32]), PTSD symptom severity (UCLA PTSD Reaction Index [33]) and depression (Short Mood and Feelings Questionnaire [34]; SMFQ). Additionally, participants completed these same measures of PTSD and depression symptom severity prior to each therapy visit.

### TF-CBT

TF-CBT was delivered by two postdoctoral clinical psychology fellows and a predoctoral clinical psychology intern. The therapists were trained in TF-CBT according to an established protocol approved by Anthony Mannarino, Ph.D., a co-developer of TF-CBT, which included completion of TF-CBT*Web* (accessible at www.musc.edu/tfcbt) an online TF-CBT training, three days of in-person TF-CBT training with Dr. Mannarino, and one hour of weekly supervision with a licensed clinical psychologist with expertise in supervising the model. TF-CBT in this study used a 12-week protocol of 60 to 90 minute weekly sessions.

### MRI Acquisition

Among the treatment-seeking sample, a Philips 3T Achieva X-series MRI system with a 32-channel head coil (Philips Healthcare, USA) was used to acquire imaging data. Anatomic images were acquired with a MPRAGE sequence (matrix = 256x256, 160 sagittal slices, TR/TE/FA = 2600ms/3.02ms/8^0^, final resolution = 1x1x1mm^3^ resolution). Echo planar imaging (EPI) sequences were used to collect the functional images using the following sequence parameters: TR/TE/FA = 2000ms/30ms/90^0^, FOV = 240x240mm, matrix = 80x80, 37 oblique slices (parallel to AC-PC plane to minimize OFC sinal artifact), slice thickness = 2.5 mm with a 0.5 mm gap between slices, resampled during preprocessing to a final resolution = 3x3x3 mm^3^.

The healthy control participants were recruited for a separate study and their imaging acquisition parameters were slightly different. An 8-channel head coil was used to acquire the imaging data. Anatomic images were collected using identical sequences and parameters. The EPI images were collected using identical parameters except slice thickness was 3mm and collected with an interleaved sequence. The head coil differences between groups is a limitation of the current study; nonetheless, it worth noting that large fMRI repositories combine datasets across scanners, head coils, and acquisition parameters under the premise that variability in scan parameters exerts less of an impact on brain function estimates relative to interindividual differences [35,36].

### Image preprocessing

Image preprocessing followed standard steps and was completed using AFNI software. In the following order, images underwent despiking, slice timing correction, deobliquing, motion correction using rigid body alignment, alignment to participant’s normalized anatomical images, spatial smoothing using a 8 mm FWHM Gaussian filter (AFNIs 3dBlurToFWHM that estimates the amount of smoothing to add to each dataset to result in the desired level of final smoothing), and rescaling into percent signal change. Images were normalized using the MNI 152 template brain. Following recent recommendations [37,38], we corrected for head motion-related signal artifacts by using motion regressors derived from Volterra expansion, consisting of [R R^2^ R_t-1_ R^2^_t-1_], where R refers to each of the 6 motion parameters, and separate regressors for mean signal in the CSF and WM. This step was implemented directly after motion correction and normalization of the EPI images in the image preprocessing stream. Additionally, we censored TRs from the first-level GLMs based on a previously used threshold of framewise displacement (FD) > 0.5. FD refers to the sum of the absolute value of temporal differences across the 6 motion parameters; thus, a cut-off of 0.5 results in censoring TRs where the participant moved, in total across the 6 parameters, more than ~0.5 mm plus the immediately following TR (to account for delayed effects of motion artifact). Additionally, we censored isolated TRs where the preceding and following TRs were censored, and we censored entire runs if more than 50% of TRs within that run were censored.

### Implicit Threat Processing Task

During this commonly used task [39], participants made button presses indicating decisions related to the sex of the poser while viewing human faces taken from the NimStim facial stimuli set. The faces contained either neutral or fearful expressions, presented either overtly or covertly, in alternating blocks. There were an equal number of female and male faces. Overt faces were presented for 500 ms, with a 1200 ms inter-stimulus-interval displaying a blank screen with a fixation cross, in blocks of 8 presentations for a total block length of ~17 s. Covert face blocks used a similar design but were presented for 33 ms followed immediately by a neutral facial expression mask for 166 ms from the same actor depicted in the covert image, and the ISI was 1500 ms. Rest blocks that displayed a blank screen with a fixation cross and lasted 10 s were additionally included. The task was presented in two runs, each lasting ~8 min, during which each block type was presented 5 times. There were 10 total blocks for each stimulus category. We conducted parallel analyses on the contrasts of covert fear vs covert neutral, overt fear vs overt neutral, and all fear vs all neutral blocks.*fMRI Data Analysis*

### Defining task-specific network properties

To characterize patterns of large-scale network organization, we used a previously defined functional brain parcellation atlas consisting of 883 unique ROIs [40]. After accounting for individual differences in spatial coverage (e.g., some participants did not have complete coverage in the cerebellum) and signal dropout (e.g., signal dropout in the OFC), 824 ROIs were retained that were shared across all participants. Rather than calculating the correlation between ROIs using the full timeseries, we are interested in task-modulated functional connectivity and accordingly focus on functional connectivity specific to each stimulus condition for each of the 824 ROIs. This was computed using the beta series method [41,42], in which a separate beta coefficient is estimated for each unique block across each voxel, resulting in 10 beta coefficients for each voxel for each stimulus condition. This was completed using AFNI’s 3dLSS and censoring out TRs where participants moved > .5mm (see above preprocessing section). It is relevant to mention that prior research demonstrates the validity of the beta series method for block tasks and with 10 repetitions per stimulus condition [42]. By contrast, identifying a unique beta coefficient for each face presentation within each block would necessarily create collinearity within the design matrix (e.g., the stimuli aren’t jittered or randomized within a block) and considerably biased estimates. We then extracted the mean timeseries of beta coefficients across the voxels within each of 824 ROIs, separately for each stimulus condition. These series of beta coefficients were then correlated separately for each stimulus condition, resulting in four 824x824 square correlation matrices. The correlation matrices were then r-to-z transformed to improve normality, the diagonals were removed, and we restricted the matrices to positive connectivity values (i.e., ignoring anti-correlated connections that have ambiguous functional interpretations) [22].

Next, network indices were calculated on each of the connectivity matrices separately. We used the Brain Connectivity Toolbox [22] implemented in Matlab to calculate modularity (the ‘community_louvain.m’ function with a gamma value of 1.1^1^), assortativity (‘assortativity.m’), and global efficiency (‘efficiency_wei.m’). Note that we calculated these indices on the weighted functional connectivity matrix, as opposed to a binary adjacency matrix that requires arbitrary thresholding. Given that modularity is estimated using an algorithm that does not necessarily converge repeatedly on identical solutions (though the variance is considerably small), we repeated each modularity estimation 10 times for each participant for each stimulus condition and used the median modularity estimate across the 10 iterations. Note that the median modularity estimate across the 10 iterations refers to the median scalar modularity Q value and not to the module to which each ROI was assigned (which would not be valid as the module number is arbitrary across iterations). This resulted in a unique network modularity Q value for each of the four stimulus block types. We additionally computed modularity Q values using the identical community detection algorithm on a randomly generated network with a degree (centrality) distribution matching the actual network (using the ‘randmio_und.m’ function).

While numerous network organization indices exist (see [22]), we focus specifically on modularity, assortativity, and global efficiency. Modularity and global efficiency (the average inverse of the shortest path length) were chosen due to their canonical implication in complex network organization and functional specialization [10,13,43]. Assortativity was chosen due to its relationship with network resilience to nodal insults [23]. Given that we are testing the relationship between network organization and treatment response, network resilience demonstrates clear face validity as a possible index of interest.

### Defining group-level community structure of the brain during emotion processing

**Fig 1:**
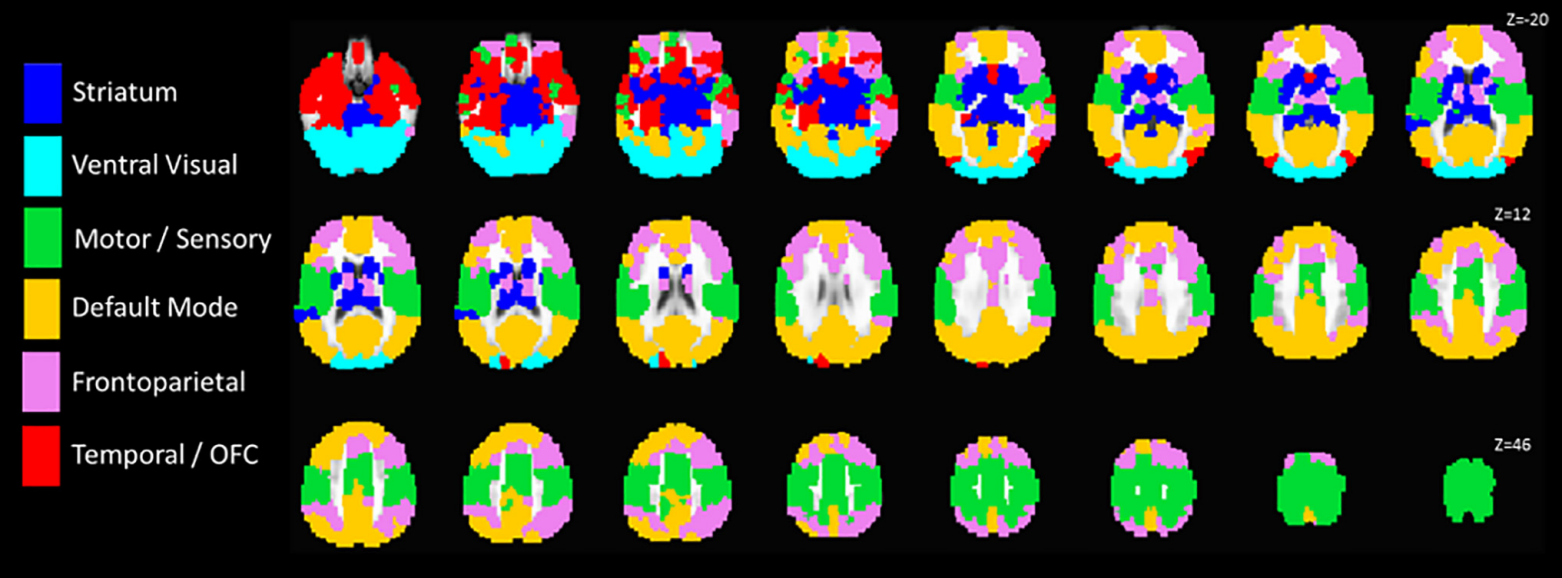
Graphical depiction of the group-level community structure. Modules were identified using the group-level median 824x824 functional connectivity matrix across all task stimuli. The modular are displayed over the ICBM 452 template brain.

To identify the specific modular organization of brain networks during emotion processing among our treatment-seeking sample, we used a similar procedure as described above, except that we calculated the square correlation matrices for each participant collapsed across stimulus categories, and then created a group-level connectivity-matrix by taking the median connectivity values across patients, resulting in a single 824x824 connectivity matrix. We then implemented the adaptation of the Louvain community detection algorithm 2000 times on this median connectivity matrix, storing the community structure (community labels of each ROI and number of detected communities) across each iteration. We then used principal component analysis (PCA) on the network memberships of the ROIs across the 2000 iterations to identify the six network modules explaining the greatest source of variance in community structure across the 2000 iterations (six networks were chosen based on the scree plot from the PCA and the median number of detected communities across the 2000 iterations). This community structure is depicted in Fig 1. Note that this analysis is meant to provide an estimate of the group’s community structure to aid in visualization and interpretation; the community detection algorithm was implemented separately on each individual when estimating Q values for subsequent analysis with treatment outcome.

### PTSD Symptom Change

Following a recent study linking fMRI data to symptom change in depression [44]and our prior analysis with this sample [45], our primary measure of clinical response consisted of slope estimates representing trajectories of PTSD symptom change across treatment sessions. We calculated these slope estimates of symptom trajectories across treatment using ordinary least squares regression: Y_t_ = B_0_ + B_1_X + B_2_Y_t-1_, where X is a linear predictor consisting of each measurement number, and Y_t-1_ is an autoregressive predictor (the clinical assessment measure at time t-1). Using a trajectory slope, as opposed to only pre-post treatment measures, has the advantage of incorporating all data points from a given subject and therefore results in more reliable estimates of change over time that are less affected by week-to-week variability in symptom severity (Heller, Johnstone, Peterson, Kolden, Kalin, & Davidson 2013). S1 Fig provides a histogram of the PTSD symptom slopes to indicate the degree of variability in the sample (also see Table 1 for mean and SD).

### Defining the relationship between network patterns and symptom reductions during TF-CBT

We used robust regression [46] analyses with a bisquare weighting function (Matlab’s robustfit) to test the degree to which stimulus-specific network patterns were related to PTSD symptom improvements. For identifying pre-treatment network predictors of subsequent PTSD symptom change, we regressed the PTSD symptom slope simultaneously onto: pre-treatment level of PTSD symptoms (controlling for initial PTSD severity), the contrast of fear vs neutral block network index (i.e., network measure for fear–network measure for neutral), and the sum of pre-treatment framewise displacement (i.e., controlling for degree of head motion, which can affect functional connectivity estimates). Separate analyses were conducted for each of the three network indices and for covert and overt stimulus blocks.

For identifying pre- to post-treatment changes in network patterns that correlated with pre- to post-treatment changes in PTSD symptoms, we conducted comparable analyses in which the PTSD symptom slopes were regressed simultaneously onto: pre-treatment level of PTSD symptoms (controlling for initial PTSD severity), the contrast of [(post-treatment fear vs neutral block network index)–(pre-treatment fear vs neutral block network index)], and the sum of pre-treatment and post-treatment framewise displacement.

## Results

### Task engagement is associated with modular and positively assortative organization of functional brain connectivity

Across all four stimulus block types at both pre- and post-treatment, the estimated network modularity Q values across the participants were significantly greater than zero (all *p*s < .001) and significantly greater than a randomly generated network of the same size with similar degree distribution (all *p*s < .001) (S2 Fig). Similarly, network assortativity coefficients were significantly positive and greater across tasks than a randomly generated network (all *p*s < .001) (S3 Fig). By contrast, global network efficiency was significantly lower than a comparable randomly generated network across all stimulus conditions (all *p*s < .001) (S4 Fig). These data confirm that task engagement is associated with increased modular and resilient organization of brain functional connectivity while simultaneously decreasing global functional integration.

Fig 1 illustrates the estimated community structure from the median functional connectivity matrix across all participants at pre-treatment. As can be seen, the functional connectivity matrix illustrates a brain organization pattern of distinct and spatially distributed communities representing a frontoparietal network, striatal network, ventral visual stream network, motor and sensory network, default mode network, and temporal lobe and orbitofrontal cortex network. For illustrative purposes, S5 Fig demonstrates the effect of individual differences in estimated modularity Q values on the functional connectivity patterns between networks. As can be seen, participants with high Q values (using a median split across all stimuli) have more clearly segregated patterns of functional connectivity between the networks compared to the participants with low Q values. This visually demonstrates the effect of individual differences in Q values estimates of modularity on the global patterns of functional connectivity.

### Relationship between brain network organization and PTSD symptom reduction

**Fig 2:**
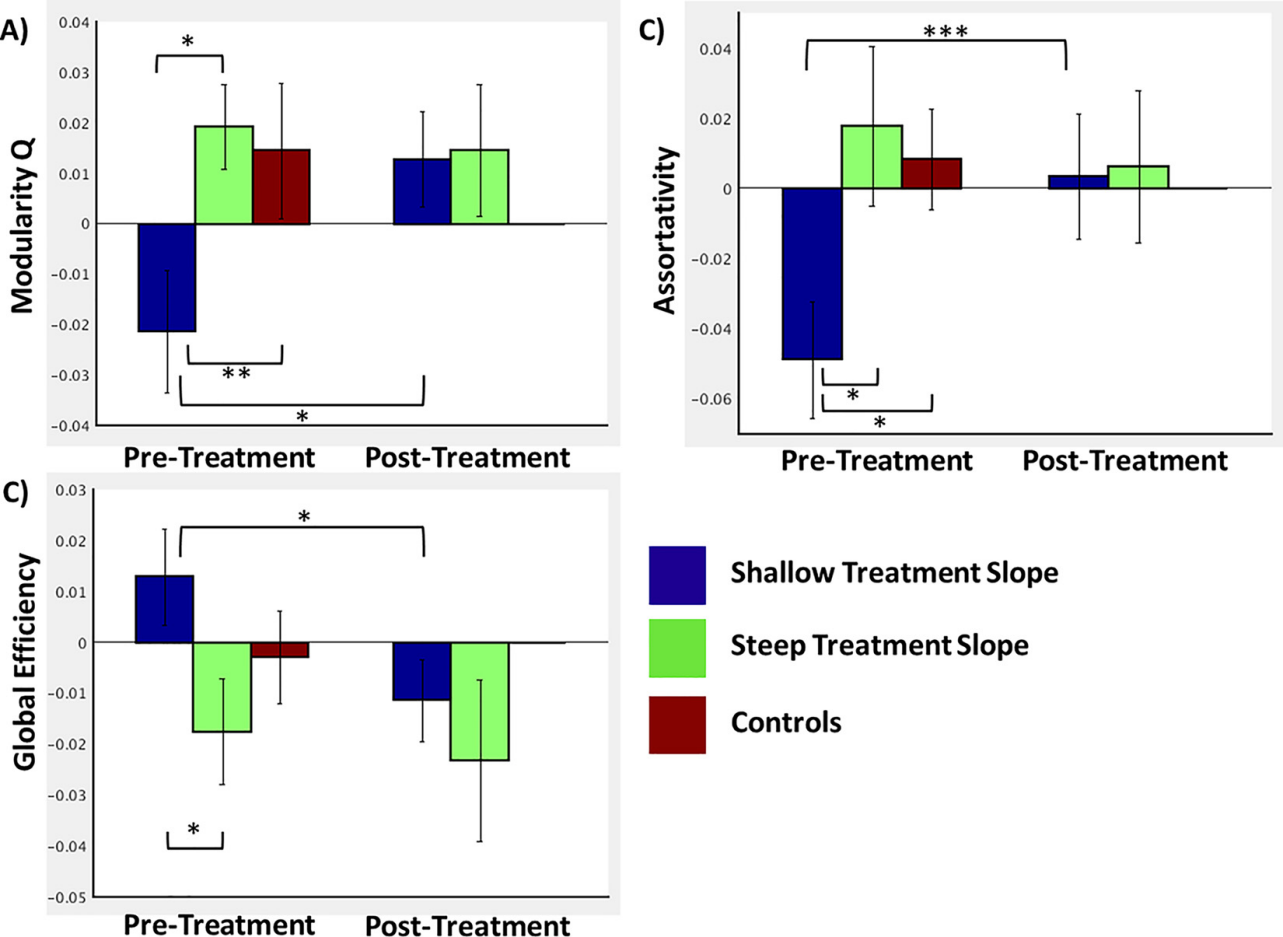
Bar graphs comparing the network organization indices of modularity (panel A), global efficiency (panel B), and assortativity (panel C) between patients with steep (n = 10) and shallow (n = 10) treatment responses. The pre-treatment comparisons additionally include the mean network indices for a healthy comparison group of 15 adolescent girls. Error bars indicate standard errors. * indicates *p* < .05; ** indicates *p* = .076; *** indicates *p* = .059.

Robust regression analyses demonstrated that the pre-treatment covert fear vs covert neutral modularity Q value contrasts significantly predicted PTSD symptom trajectory slopes (B = -.42, *t* = -2.69, *p* = .016) when controlling for initial PTSD symptom severity and head motion (Fig 2), such that participants with steeper treatment slopes had greater pre-treatment modularity compared to participants with shallower slopes. Comparable analyses also demonstrated that the global efficiency contrast for covert fear vs neutral blocks significantly predicted PTSD symptom trajectory slopes (B = .53, *t* = 3.16, *p* = .006), as did the covert fear vs neutral contrast for the assortativity coefficient (B = -.23, *t* = -2.25, *p* = .039), indicating that participants with steeper treatment slopes had lower efficiency and higher assortativity at pre-treatment. The comparable analyses for the overt fear vs neutral contrasts for modularity, global efficiency, and assortativity were all non-significant (all *ps* > .4).

**Fig 3:**
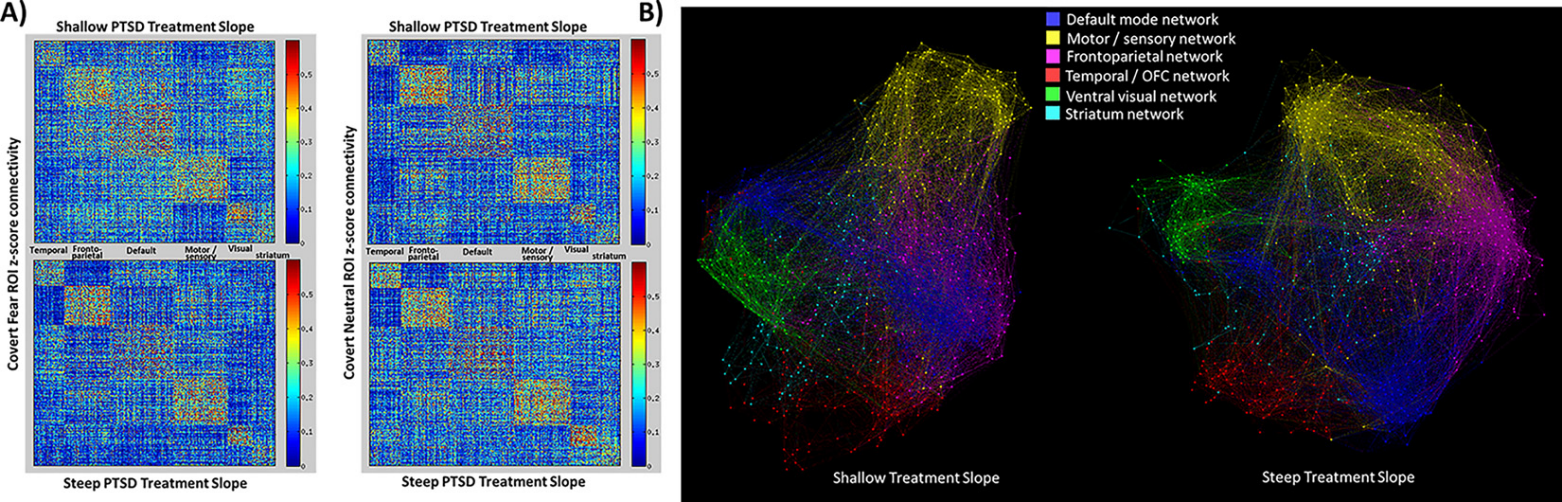
A) Heat map comparison of the median 824x824 functional connectivity matrices of participants with steep (n = 10) and shallow (n = 10) treatment slopes for pre-treatment covert fear blocks (left) and cover neutral blocks (right). B) Visual depiction of covert fear network organization (from the left hand column in Panel A) for the participants with steep (n = 10) and shallow (n = 10) treatment slopes. The figures were generated using Gephi (v0.8.2) with the ForceAtlas2 graph layout algorithm using group-level connectivity matrices with a density threshold of 0.02.

To illustrate these effects on the functional connectivity patterns, we displayed the mean pre-treatment functional connectivity maps for adolescents with steep vs shallow PTSD symptom trajectory slopes (via a median split) during TF-CBT separately for covert fear and covert neutral blocks (Fig 3, left hand portion). As can be seen, and consistent with the direction of the statistical relationships reported in the above paragraph, participants with shallow PTSD symptom trajectory slopes demonstrated less modular organization of functional connectivity relative to participants with steep PTSD symptom trajectory slopes only for covert fear blocks. An alternative visual representation of network organization differences is additionally indicated in the right hand portion of Fig 3, where the differences in network segregation are also clearly visible between the groups.

Testing whether changes in modularity across treatment are associated with PTSD symptom trajectory slopes, robust regression analyses demonstrated that change in assortativity from pre-to post-treatment for the covert fear vs covert neutral contrast significantly predicted PTSD symptom trajectory slopes (B = .17, *t* = 2.32, *p* = .035), controlling for initial PTSD symptom severity and head motion. Comparable analyses demonstrated a marginally significant relationship between pre- to post-treatment changes in the covert fear vs neutral contrasts and PTSD symptom slopes for modularity (B = .29, t = 2.09; p = .054), but not global efficiency (B = -.21; *t* = -1.59; *p* = .13). The comparable analyses using the overt fear vs neutral contrasts were not significant (*ps* > .5). However, subsequent analyses demonstrated that only the participants with shallow courses of symptom trajectories demonstrated significant pre- to post-treatment changes in global efficiency (*t* = 2.4, *p* = .04), modularity (*t* = 2.62, *p* = .028), and marginally significant changes in assortativity (*t* = 2.16, *p* = .059), while the participants with steep symptom slopes did not (*ps* > .75) (see Fig 2).

### Brain Organization Compared to Healthy Controls

As can be seen in Fig 2, at pre-treatment, adolescents with steep symptom trajectory slopes generally more closely resembled the healthy control participants. The healthy control participants demonstrated significantly greater assortativity than PTSD participants with shallow treatment slopes (*t* = 2.57, *p* = .017), marginally significantly greater modularity (*t* = 1.86, *p* = .076), and there were no differences in global efficiency (*t* = 1.17, *p* = .25). Further, the group contrast of [PTSD participants with steep trajectories + healthy controls] versus PTSD participants with shallow trajectories was significant for both modularity (*t* = 2.41, *p* = .022) and assortativity (*t* = 2.76, *p* = .009) and marginally significant for global efficiency (*t* = 1.75, *p* = .09).

### Addressing potentially confounding variables

Given variability among participants in age, psychotropic medication use, comorbid diagnoses, and ethnicity, we conducted additional robust regression analyses to test for relationships between these variables and treatment response (PTSD symptom slopes), modularity, assortativity, and global efficiency for the pre-treatment contrast of covert fear vs covert neutral. The potentially confounding variables did not demonstrate significant relationships with treatment response (all *ps* > .18) or the network organization indices (all *p*s > .17). Finally, given that the primary analyses reported above tested the relationship between network organization and treatment response while controlling for only pre-treatment PTSD severity and head motion, we repeated the analyses testing whether the relationships between network organization and treatment response held when separately controlling for age, medication, verbal IQ, number of comorbid diagnoses, and ethnicity. These analyses demonstrated essentially no effect of the different possible confounding variables on the magnitude of the relationship between the network organization indices and treatment response (see S6 Fig).

## Discussion

The purpose of the present investigation was to identify modes of functional connectivity of large-scale brain networks associated with individual variation in PTSD symptom reduction during TF-CBT among adolescent girls with assault-related PTSD. It is important to acknowledge that our design did not include a no-treatment control group, precluding the ability to differentiate treatment-specific symptom reduction vs spontaneous symptom reduction, and this limitation should temper inferences accordingly. With that limitation clearly stated, we summarize the major findings as follows. First, we demonstrated that the large-scale network organization indices of modularity, assortativity, and global efficiency all differed significantly from random networks (S3–S5 Figs), providing support for the validity of these indices applied to task data. Second, we found that the response at pre-treatment to implicit fear processing for the network indices of modularity, global efficiency, and assortativity predicted greater subsequent PTSD symptom reduction. Third, we found that the pre-treatment patterns of functional network organization among the adolescents who responded best to therapy more closely resembled the functional network organization patterns of healthy controls, whereas the functional network organization patterns of the adolescents who did not respond as well to treatment tended to differ from the normative patterns. Fourth, we observed that it was specifically the subgroup of adolescents who responded *less* to treatment that demonstrated the greatest pre- to post-treatment changes in functional network organization. Finally, the observed effects were specific to covert fear processing and not observed for overt fear processing, suggesting the primacy of automatic/implicit processing rather than strategic/explicit processing of fear signals.

The finding that pre-treatment patterns of implicit fear processing network organization predicted subsequent PTSD symptom reduction during therapy suggests that these patterns represent neural mechanisms that enable subsequent symptom reduction. Specifically, the extent to which the network-level organization of neural information processing as a response to implicit perceptions of fear for adolescent females with assault-related PTSD conformed to the normative pattern of simultaneously enhancing modular and assortative organization and decreasing global efficiency appears to signal symptom reduction. As noted above, modular organization of complex networks decreases wiring costs while concurrently enhancing dynamic stability, and networks with higher assortativity are more resilient and less susceptible to targeted node insults [16,18,22]. Global efficiency refers to the average shortest path lengths within a network, with higher global efficiency representing a more integrated network but with higher wiring costs [22]. As such, girls who demonstrated the greatest symptom reduction functionally organized brain networks in response to covert fear signals in a manner characterized by greater resilience and modularized information processing but with lower overall efficiency. By contrast, girls who demonstrate the lowest symptom reduction organized brain networks in response to covert signals in a manner characterized by greater overall network integration but at the cost of less resilient and modularized organization. It is noteworthy that the healthy control adolescent girls more closely resembled the PTSD participants with better symptom reduction. That is, it is not the case that the girls who improved most had some type of enhanced, compensatory, or overdeveloped network organizational pattern; rather, it seems to be the case that the girls who responded most poorly to therapy lacked normative patterns of brain organization prior to engaging in therapy. It is certainly the case that differing head coils used between the healthy group and the PTSD group should temper inferences from the direct statistical comparisons of the groups; nonetheless, there is no reason to suspect that the head coil used would alter the qualitative direction of the effects which corroborate the differing network patterns in the girls with shallower treatment slopes.

The maturation of cognitive ability across adolescence is related to the dynamic maturation of network modules as cognitive systems with their increasing functional segregation [47]. Perhaps a varying response to trauma is the extent to which this developmental process of growing network modularity is developmentally disrupted by trauma exposure rendering some girls less readily amenable to the therapeutic effects of TF-CBT on network organization and trauma recovery. A further hypothesis is that intact network organization responses enable neuroplasticity necessary for cognitive processing of the traumatic memory and related therapeutic learning that occurs during TF-CBT. It is also noteworthy that the effects were only found for covert fear signals. Perhaps strategic processing of overt fear signals washes out differences in network organization across individuals, whereas covert fear signals, which preclude the use of a cognitive strategy, allow these inherent network organization differences to emerge.

While the finding that pre- to post-treatment increases in assortativity, and to a lesser extent modularity, during covert fear processing scaled with PTSD symptom reduction suggests that, overall, symptom reduction might be mediated by increased assortative brain network organization, it is important to note that in fact it was only the girls with the least symptom reduction who demonstrated pre- to post-treatment changes in modularity, global efficiency, and assortativity. By contrast, the girls with steeper symptom slopes did not demonstrate significant pre- to post-treatment changes in network organization. This observation precludes the inference that symptom reduction is mediated by changes in large-scale network organization because the girls with the least symptom reduction demonstrated the greatest normalization of network organization. As such, these observations are consistent with the hypothesis that intact normative brain organization responses to covert fear signals represents a mechanism *enabling* subsequent symptom reduction during therapy, as opposed to being a mechanism of symptom reduction itself. That is, these data suggest that it might be necessary for the brain to attain normative modes of network organization, characterized by modular and resilient organization, before symptom reduction can then occur. This causal relationship was not tested here yet could directly be tested through repeated measurement of brain organization and PTSD symptoms throughout treatment (e.g., PTSD symptom assessment and fMRI after every 3 sessions for 12–18 sessions). Future research is clearly necessary to elucidate the temporal relationships between large-scale patterns of brain organization, their changes during treatment, and their relationships with trauma recovery.

The observation that a (relatively speaking) homogenous group of adolescent girls with PTSD demonstrated significant individual differences in these large scale brain organization patterns, with only the girls with steeper treatment slopes demonstrating normative patterns, underscores the inference that these organization patterns are not a mechanism of PTSD. Rather, brain networks with modular and resilient organization are perhaps more dynamic, thus permitting new learning during therapy and subsequent symptom reduction. It is also interesting to observe that TF-CBT had opposing effects on participants depending on pre-treatment modes of network organization: among those with intact normative network organization, TF-CBT appeared to produce clinically significant PTSD symptom reduction (mean decrease of 20 points on the UCLA-PTSD index) without modifying network organization; among those with altered network organization, TF-CBT appeared to normalize the network alterations while producing less PTSD symptom reduction (mean decrease of 6 points on the UCLA PTSD index). These findings suggest interesting approaches to individualize and optimize clinical interventions. If it were the case that network normalization enables symptom reduction, then we would expect that the low-responding group would have begun demonstrating greater PTSD symptom reductions had therapy continued. It is interesting then to speculate whether there are components of TF-CBT that specifically enable network normalization (e.g., the relaxation and affective modulation skills) and components that specifically enable symptom reduction (e.g., cognitive processing of the trauma). If this is the case, then it might advantageous to prolong those components that enable network normalization among those without normative brain organization at pre-treatment until the patterns attain a normalized brain state. By contrast, among those with normative brain organization patterns, it might be possible to move more quickly into the treatment components that target symptom reduction. However, it is also possible that the same TF-CBT component elicit different responses depending on the adolescent’s maturational state of brain organization and resulting cognitive plasticity. Future research is clearly needed along these lines, preferably with a design in which symptoms and brain networks are repeatedly measured across a longer duration of therapy (e.g., up to 18 sessions based on participant need; [48]).

While the current data provide novel inferences regarding large-scale brain network organization patterns in adolescent PTSD and their relationships with symptom reduction during TF-CBT, the current study is not without limitations. First, the sample was small and limited to girls, necessitating replication with larger and mixed-sexed or male samples. Second, we did not have a control group of adolescents with PTSD who did not receive treatment and were assessed at matched intervals to our treatment group, therefore we cannot differentiate between spontaneous symptom courses of reduction vs response to treatment specifically. Third, brain imaging data were only acquired at pre- and post-treatment, precluding inferences regarding causal relationships (e.g., did the brain patterns or PTSD symptoms change first?). Fourth, follow-up data were not available, precluding inferences regarding maintenance of either brain changes or PTSD symptom reductions. Fifth, the healthy control group was scanned using a different head coil (8-channel vs 32-channel among the PTSD sample), introducing the possibility of head-coil induced confounds on the network indices among this sample, and statistical comparisons between the groups in network organization should accordingly be interpreted with caution. Sixth, we did not exclude based on current psychotropic medication usage, and while we did not find a relationship between medication usage and treatment response or network indices, it is nonetheless possible that medication usage confounded the results in some unforeseen manner. Seventh, we did not include a manipulation check on the masking of the stimuli to conclusively verify that participants were unable to explicitly process the masked faces. Finally, we did not have a control group of abused adolescents who did not have a current diagnosis of PTSD, thus inferences cannot be made regarding differential effects of PTSD vs trauma exposure in this study.

## Supporting Information

S1 Fig Histogram of log-transformed PTSD symptom slopes for the current sample. (TIF)

S2 Fig Comparison of mean modularity values across the stimulus conditions between real networks and randomly generated networks at pre- and post-treatment. Error bars denote standard errors.
(TIF)

S3 Fig Comparison of mean global efficiency values across the stimulus conditions between real networks and randomly generated networks at pre- and post-treatment. Error bars denote standard errors.
(TIF)

S4 Fig Comparison of mean assortativity values across the stimulus conditions between real networks and randomly generated networks at pre- and post-treatment. Error bars denote standard errors.
(TIF)

S5 Fig Heat map comparison of the high (n = 10) and low (n = 10) modularity (Q value) participants’ median 824x824 functional connectivity matrix, based on a median split of Q values, at pre-tx (left hand portion) and post-tx (right hand portion). (TIF)

S6 Fig Graphical depiction of the change in beta values (top) and p values (bottom) when including different covariates in the regression models. ‘Original’ = beta values and p values for the network indices in the regression model reported in the manuscript in which head motion and pre-treatment PTSD symptom severity are included in the model. ‘Age’ = beta values and p values for the network indices in the regression model when also including age as a covariate, and so forth for ‘race’, ‘IQ’, etc. ‘Meds’ = psychiatric medication. ‘comorbid’ = number of comorbid diagnoses.
(TIF)
