# Supplementary figures and images for: Modes of Large-Scale Brain Network Organization during Threat Processing and Posttraumatic Stress Disorder Symptom Reduction during TF-CBT among Adolescent Girls
Source: PLoS One. 2016 Aug 9;11(8):e0159620. doi: 10.1371/journal.pone.0159620 (PMC4978452; doi:10.1371/journal.pone.0159620)

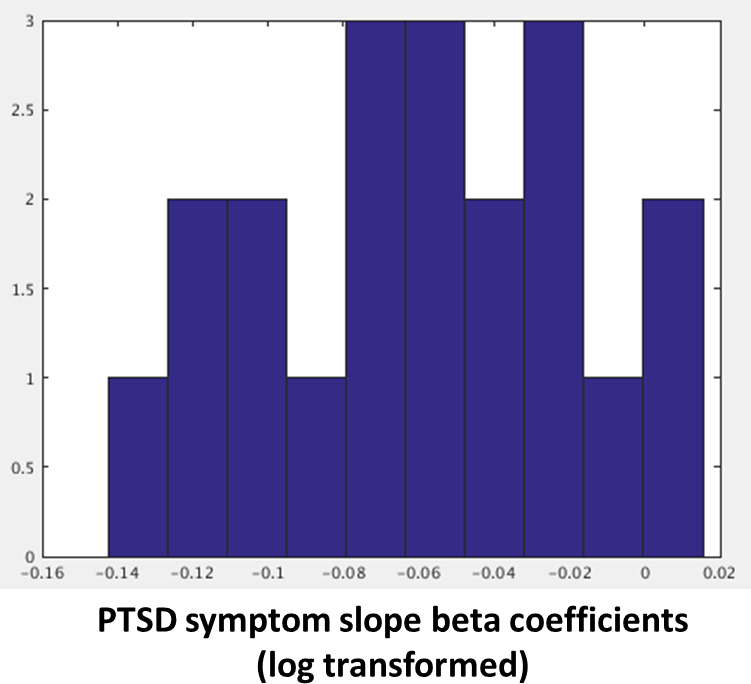

Supplement: S1 Fig — (TIF) [file pone.0159620.s001.tif]

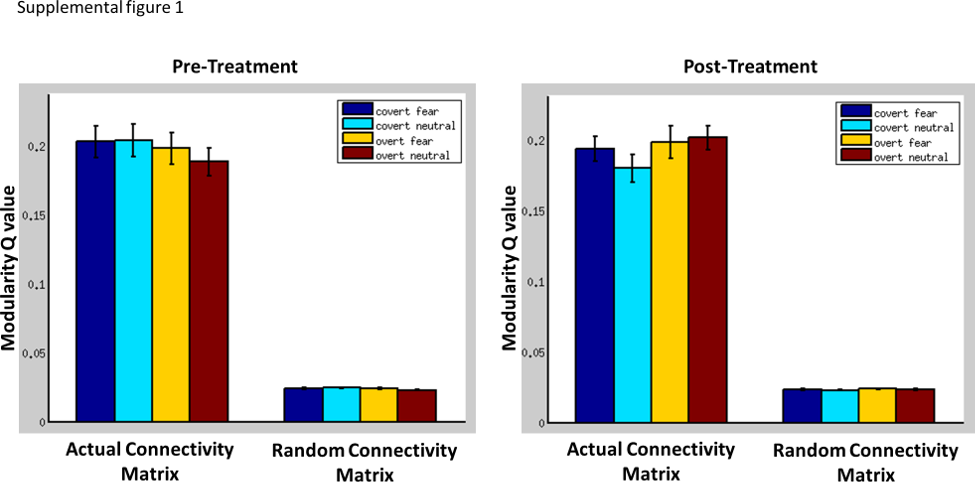

Supplement: S2 Fig — Error bars denote standard errors. (TIF) [file pone.0159620.s002.tif]

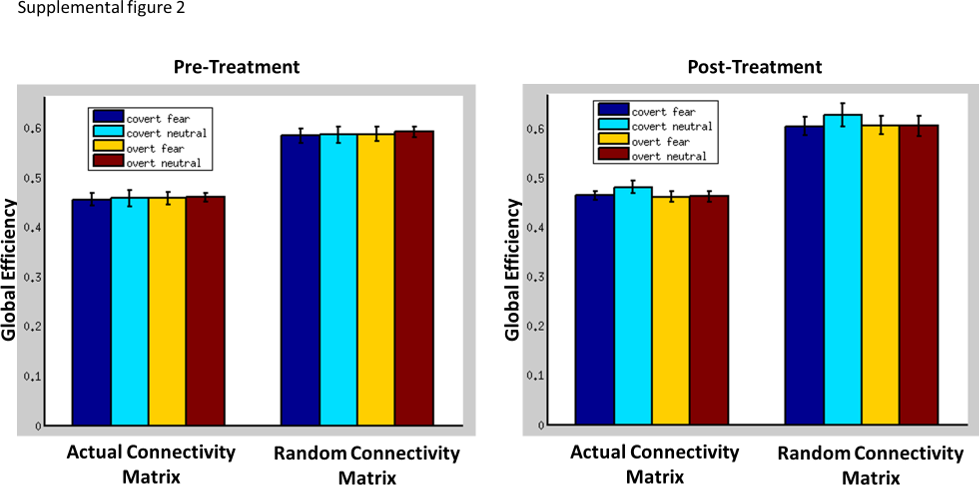

Supplement: S3 Fig — Error bars denote standard errors. (TIF) [file pone.0159620.s003.tif]

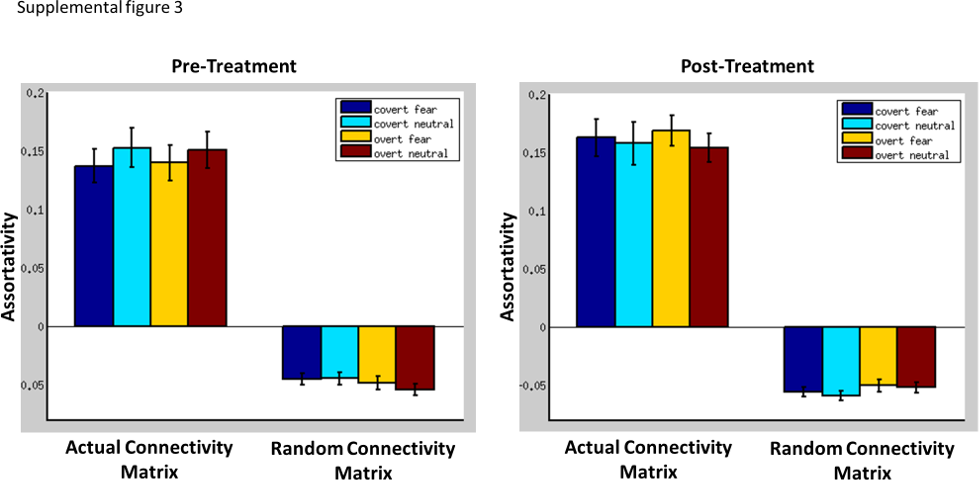

Supplement: S4 Fig — Error bars denote standard errors. (TIF) [file pone.0159620.s004.tif]

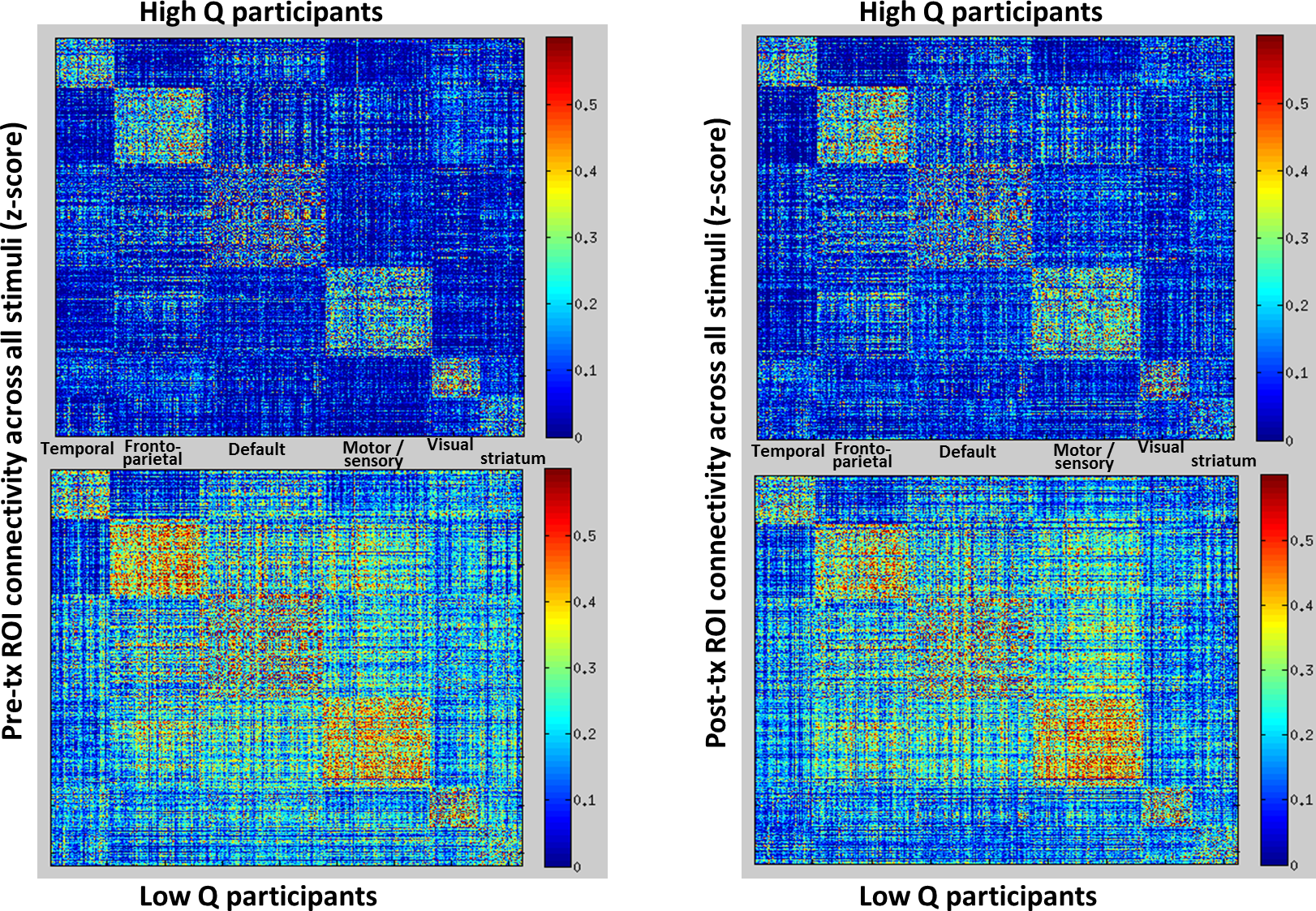

Supplement: S5 Fig — (TIF) [file pone.0159620.s005.tif]

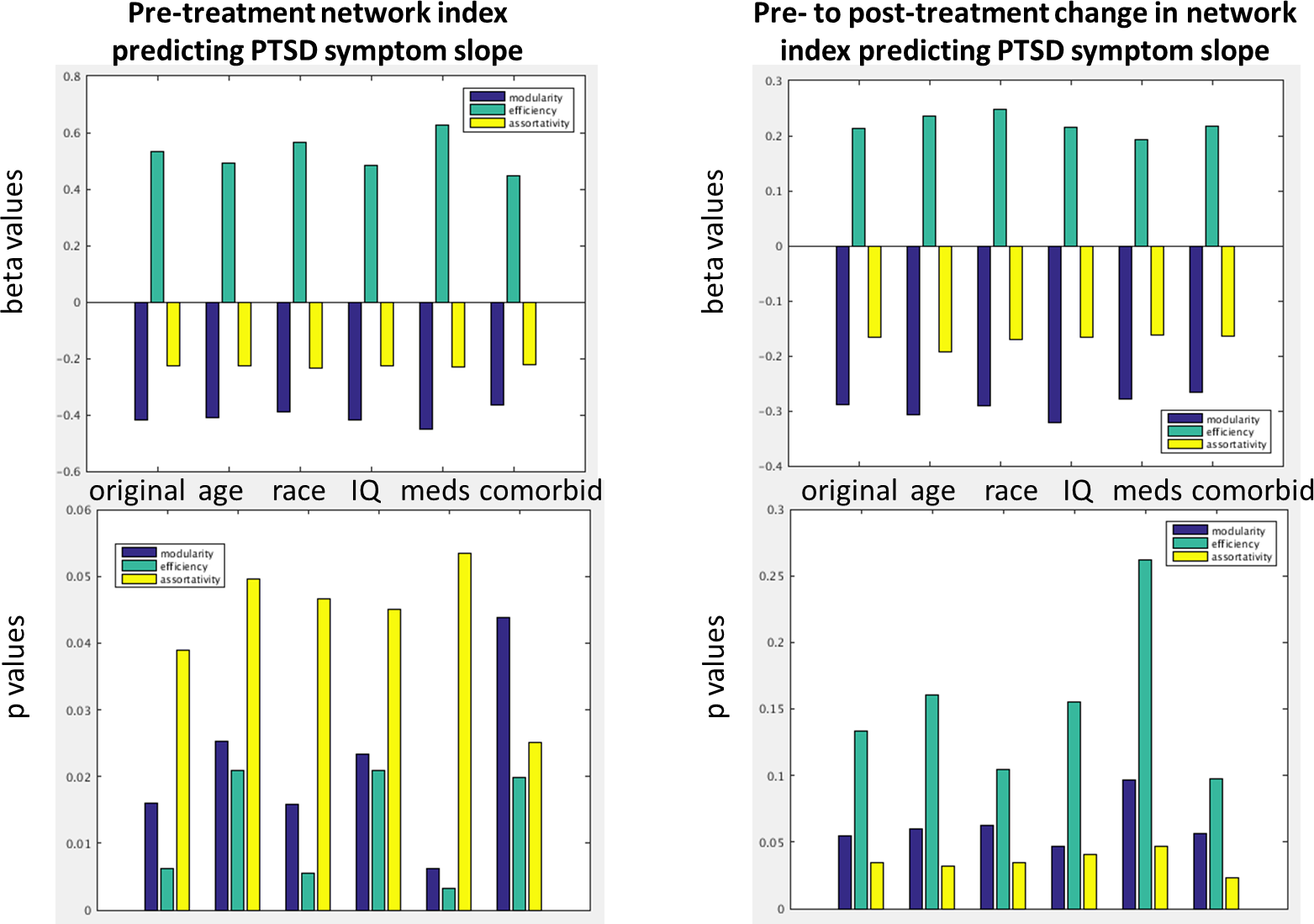

Supplement: S6 Fig — Graphical depiction of the change in beta values (top) and p values (bottom) when including different covariates in the regression models. ‘Original’ = beta values and p values for the network indices in the regression model reported in the manuscript in which head motion and pre-treatment PTSD symptom severity are included in the model. ‘Age’ = beta values and p values for the network indices in the regression model when also including age as a covariate, and so forth for ‘race’, ‘IQ’, etc. ‘Meds’ = psychiatric medication. ‘comorbid’ = number of comorbid diagnoses. (TIF) [file pone.0159620.s006.tif]
